# Virological investigation of four outbreaks of influenza B reassortants in the northern region of Taiwan from October 2006 to February 2007

**DOI:** 10.1186/1756-0500-2-86

**Published:** 2009-05-09

**Authors:** Yuan-Ming Lee, Sheng-Fan Wang, Cheng-Ming Lee, Kuan-Hsuan Chen, Yu-Jiun Chan, Wu-Tse Liu, Yi-Ming Arthur Chen

**Affiliations:** 1 Institute of Public Health, School of Medicine, National Yang-Ming University, Taipei, Taiwan, ROC; 2 Division of Clinical Virology, Department of Pathology and Laboratory Medicine, Taipei Veterans General Hospital, Taipei, Taiwan, ROC; 3 Division of Laboratory Medicine, National Yang-Ming University Hospital, Yilan, Taiwan, ROC; 4 AIDS Prevention and Research Center, National Yang-Ming University, Taipei, Taiwan, ROC; 5 Institute of Medical Technology, National Yang-Ming University, Taipei, Taiwan, ROC; 6 Division of Infectious Disease, Department of Medicine, Taipei Veterans General Hospital, Taipei, Taiwan, ROC; 7 Department of Microbiology, School of Medicine, National Yang-Ming University, Taipei, Taiwan, ROC

## Abstract

**Background:**

From October 2006 to February 2007, clinical specimens from 452 patients with symptoms related to respiratory tract infection in the northern region of Taiwan were collected. Real-time PCR and direct immunofluorescent antibody tests showed that 145 (32%) patients had influenza B virus infections. Subsequently, nucleotide sequence analyses of both hemagglutinin (HA) and neuraminidase (NA) genes of 39 isolates were performed. Isolated viruses were antigenically characterized using hemagglutinin inhibition (HI) test.

**Findings:**

Phylogenetic tree analysis showed that all the isolates belonged to the B reassortant lineage with HA gene belonged to the B/Victoria/2/87 lineage and the NA gene belonged to the B/Yamagata/16/88 lineage. In addition, a group of children aged between 6 to 8 years old resided in Yilan county were infected with a variant strain. Hemagglutinin inhibition (HI) tests confirmed that all the reassortant influenza B viruses were B/Malaysia/2506/04-like viruses. Pre- and post-immunized serum samples from 4 normal volunteers inoculated with 2007 influenza vaccine were evaluated for their HI activity on 6 reassortant B isolates including two variants that we found in the Yilan county. The results demonstrated that after vaccination, all four vaccinees had at least 4-fold increases of their HI titers.

**Conclusion:**

The results indicate that the 2006–2007 seasonal influenza vaccine was effective in stimulating protective immunity against the influenza B variants identified in Yilan county. Continuous surveillance of emerging influenza B variants in the northern region of Taiwan is important for the selection of proper vaccine candidate in the future.

## Background

Enveloped orthomyxoviruses have segmented negative-sense RNA genomes that facilitate new strain development via mutations and the reassortment of gene segments. This genetic instability is responsible for annual international epidemics and periodic pandemics of influenza infections [1,2]. Influenza viruses are spread by small airborne droplets expelled while talking, breathing, and coughing. The most susceptible population is children, and school-age children are the most common spreaders of infection [3-7]. Vaccines are thought to be the most effective modality for influenza control, and phylogenetic analyses of genes of viruses provide valuable information for vaccine development and prophylaxis.

Hemagglutinin (HA) and neuraminidase (NA) genes have been used to study influenza B virus evolution [8-10]. Two lineages have been identified as co-circulating since 1983: B/Victoria/2/87 (Vic87) and B/Yamagata/16/88 (Yam88) [11], and reassortants of the two lineages have been observed since 2002 [12-18]. The increasing appearance of reassortant influenza B was reported in Taiwan from 2002 to 2005 [19].

We conducted phylogenetic analyses of HA and NA genes from influenza B virus isolates collected from a group of children complaining of severe headaches in north Taiwan between October 2006 and February 2007. Variants were further tested using a hemagglutinin inhibition (HI) test with a panel of anti-flu B antisera; our results indicate incomplete inhibition. Data from an amino acid sequence analysis of the HA protein showed greater sequence variation to B/Malaysia/2506/04-like viruses in 5 influenza B variants (B/Taiwan/3009/06, B/Taiwan/3802/06, B/Taiwan/3804/06, B/Taiwan/3805/06, and B/Taiwan/3790/06).

## Methods

### Subjects

**Figure 1:**
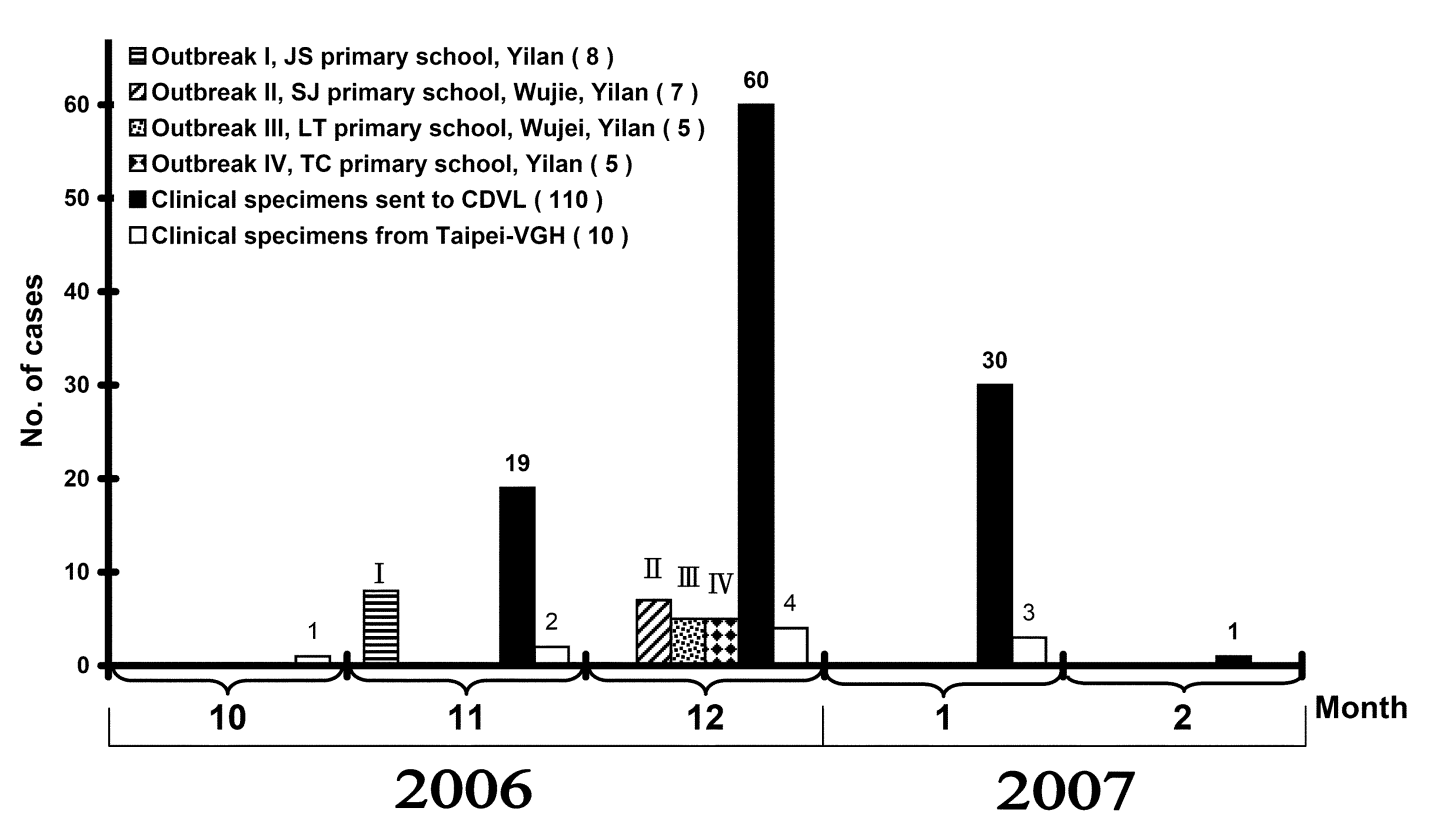
**Data on influenza B virus outbreak in North Taiwan between October 2006 and February 2007**. Of 452 clinical respiratory samples examined, 145 (32%) tested positive for a B/Malaysia/2506/04-like strain (B reassortant lineage). The data came from 4 outbreaks and 2 group samples: Outbreak I, 8 patients from Yilan, Jiaosi (YLJS); outbreak II, 7 patients from Shai-Jin, Yilan, Wujie (YLWJ-SJ); outbreak III, 5 patients from Li-Tse, Yilan, Wujie (YLWJ-LT); outbreak IV, 5 patients from Yilan, Toucheng (YLTC); group 1, 110 patients from other clinics of CDVL; group 2, 10 patients from Taipei-VGH ordinary clinic.

**Figure 2:**
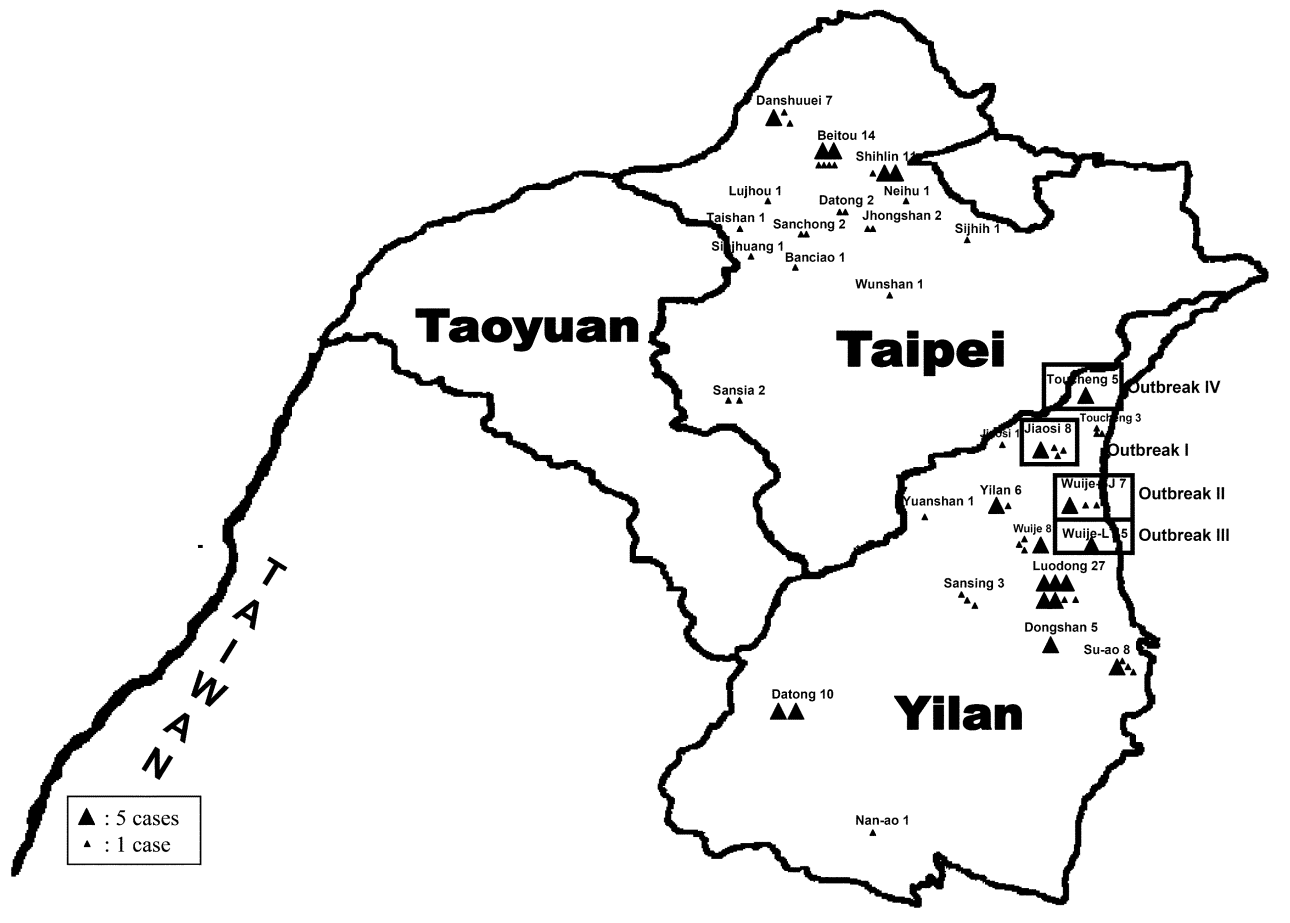
**Approximate distribution of influenza B viruses analyzed for this research**.

Between October 2006 and February 2007, 452 throat swabs from patients with symptoms related to respiratory tract infection were collected at the clinical virology laboratory of the Taipei Veterans General Hospital. This laboratory has a contract with the Taiwan's Centers for Disease Control and is responsible for the virological evaluation of all the specimens sent from different hospitals in the northern region of Taiwan. The epidemiological curve and geographic distribution were shown in Figures 1 and 2. The median age of 145 patients with influenza B infection was 8 years old, with a range from 8 days to 14 years old except that B/Taiwan/3807/06, B/Taiwan/3842/06, B/Taiwan/1575/07 and B/Taiwan/1540/07 were 19, 18, 23 and 43 years old respectively. Seventy-four patients are male and seventy-one are female. To study the hemagglutinin inhibition titers for the influenza virus B isolates, five adult volunteers including four normal individuals and one patient (P139) with impaired immune function were recruited to receive vaccines containing A/New Caledonia/20/99, A/Wisconsin/67/05 and B/Malaysia/2506/04 strains. Informed consent was obtained from all participants. Patient P139 described that he was incapable of generating anti-hepatitis B virus (HBV) surface antibodies after repeatedly inoculated with HBV vaccine. Serum samples were collected prior to and 3 months after vaccination.

### Virus Isolation and Confirmation

Filtrates from transport medium containing throat swabs were inoculated into Madin-Darby Canine Kidney (MDCK) cells. HA tests with guinea pig red blood cells were used to test the cultural supernatant for influenza B viral infection. Infected cells were detected using an IMAGEN™ influenza virus test kits (DaKo Cytomation Ltd, Cambs, UK) contain fluorescein isothiocyanate(FITC) conjugated monoclonal antibodies which bind specifically to influenza virus type B.

### RNA Extraction and RT-PCR

Viral RNA was extracted from 140 μL of infected tissue culture fluid using a QIAamp Viral RNA mini Kit (QIAGEN Inc., CA). Real-time PCR was performed with 5 μL RNA, Taqman one-step RT-PCR master mix reagents (Roche, New Jersey USA), 400 nM primer and 100 nM probe. The primers and probe used were according to Garbino et al. [20] and the cycling condition is 30 min at 48°C, 10 min at 95°C, and 40 cycles of 15 seconds at 95°C and 1 min at 60°C. In addition, 5 μL of eluted RNA were used for amplification with a QIAGEN one-step reverse transcription (RT)-PCR reagent. RT-PCR and sequencing for HA and NA were performed according to Tsai et al. [19].

### Nucleotide Sequence Analysis

DNA sequence analysis was performed using software from the Wisconsin Genetics Computer Group (GCG) [19,21,22]. All nucleotide sequences identified in this study have been deposited in GenBank (accession numbers EU234376 to EU234451).

### Phylogenetic Analysis

Alignment of the respective 739 and 251 base pairs of HA and NA gene sequences was performed using the DNASTAR MegAlign clustal method. Phylogenetic trees were constructed using the neighbor-joining method based on Kimura's 2-parameter distance matrix with 1000 bootstrap replicates, using the MEGA (version 3.0) and PHYLIP (version 3.6) software packages [23-25]. We used MEGA for construction of neighbor-joining trees and PHYLIP for parsimony and maximum-likelihood methods to verify the topology of taxas shown in the trees. In this study, all trees are shown as the neighbor-joining tree from MEGA.

### Hemagglutinin Inhibition Test

HI testing of 2006–2007 samples was performed using the World Health Organization (WHO) Influenza Reagent Kit for identifying influenza isolates, produced and distributed by WHO collaborating center for surveillance, epidemiology and control of influenza in American Continent (CDC, USA) as described in the kit's manual. Reference strain samples consisted of distinct influenza B anti-sera from four epidemics: B/Malaysia/2506/04 (B reassortant lineage), B/Hong Kong/330/01 (Victoria lineage), B/shanghai/361/02 (Yamagata lineage), and B/Sichuan/379/99 (Yamagata lineage).

## Results

### Reassortant Influenza B Virus Epidemiology

Among HA screening tests positive cases from 452 specimens, 145 (32%) were confirmed to have influenza B infection using both real-time PCR and IFA test. The epidemiological curve and geographic distribution analyses demonstrated that the influenza B infection reach the peak in December 2006 and there were four outbreaks in Yilan county located in the northern region of Taiwan: outbreak I, 8 children from Jiaosi town; outbreak II, 7 students from Shai-Jin Elementary School in Wujie town; outbreak III, 5 students from Li-Tse Elementary School in Wujie town; and outbreak IV, 5 children from Toucheng town (Figures 1 and 2). Major symptoms included vomit, rhinorrhea, myalgia, diarrhea, prostration, lethargy, pharynegeal vesicles or ulcers, cough, sore throat, headache, and fever (temperature > 38°C), with a duration of 1–2 weeks. Of the patients from Yilan county, all 25 were in kindergarten or primary school, aged between 6 and 8 years old.

### Phylogenetic Analysis

**Figure 3:**
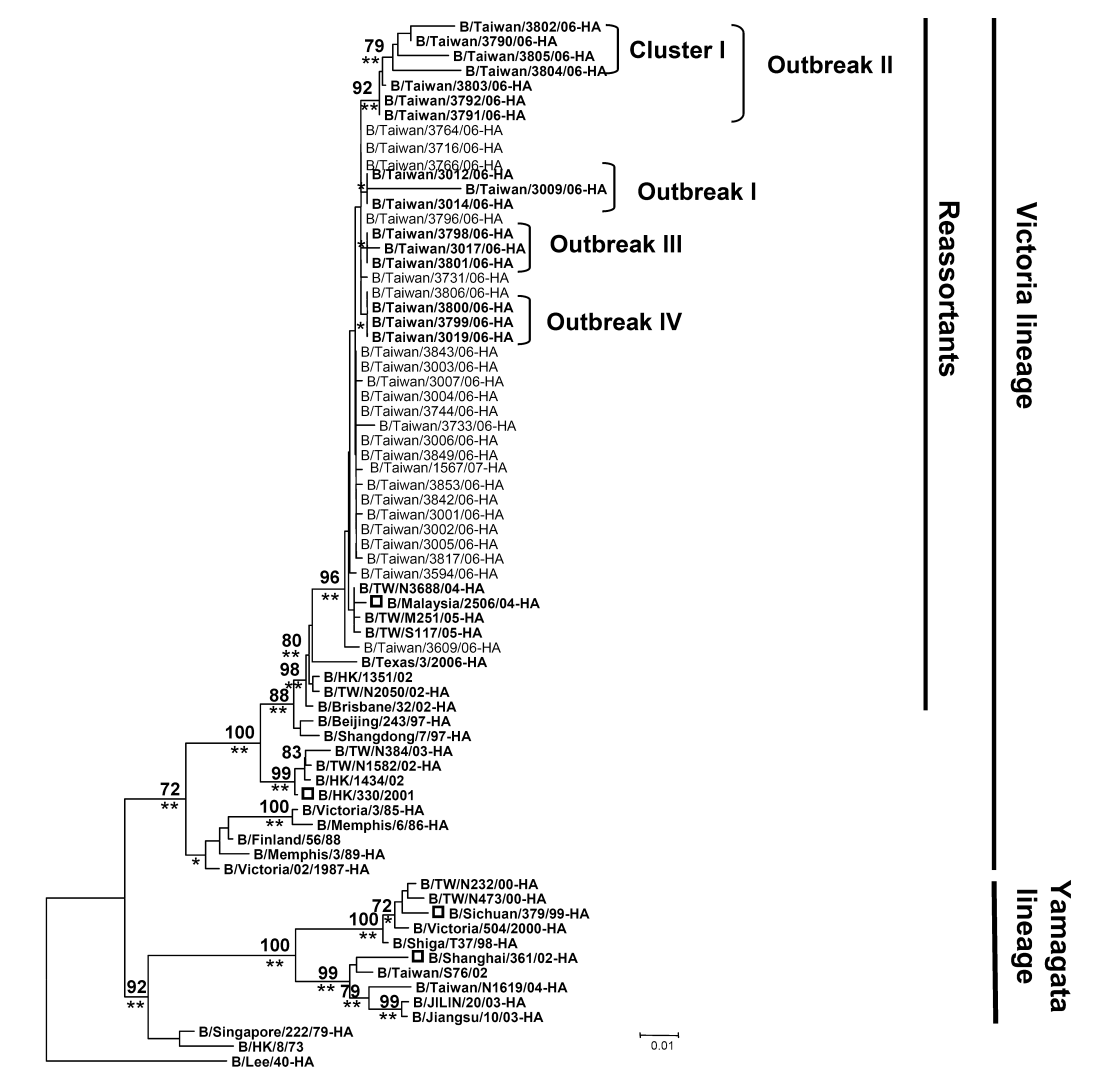
**A phylogenetic analysis of HA genes from influenza B virus isolates obtained between October 2006 and February 2007**. Dendrogram of 39 influenza B strains and 32 GenBank reference strains is based on 739 nucleotides (75 to 813) of the HA1 gene (neighbor-joining method with DNASTAR distance measure program; MEGA, Version 3.0). The linear unrooted phylogenetic dendrograms present the B/Lee/40 sequence as an outgroup. Branch length indicates genetic distance according to indicated distance scale (0.01, 1% difference). Bootstrap analysis percentages (n = 1000) for each branch are indicated. The statistical p-values were labeled on each node (*P < 0.05 and **P < 0.01).

**Figure 4:**
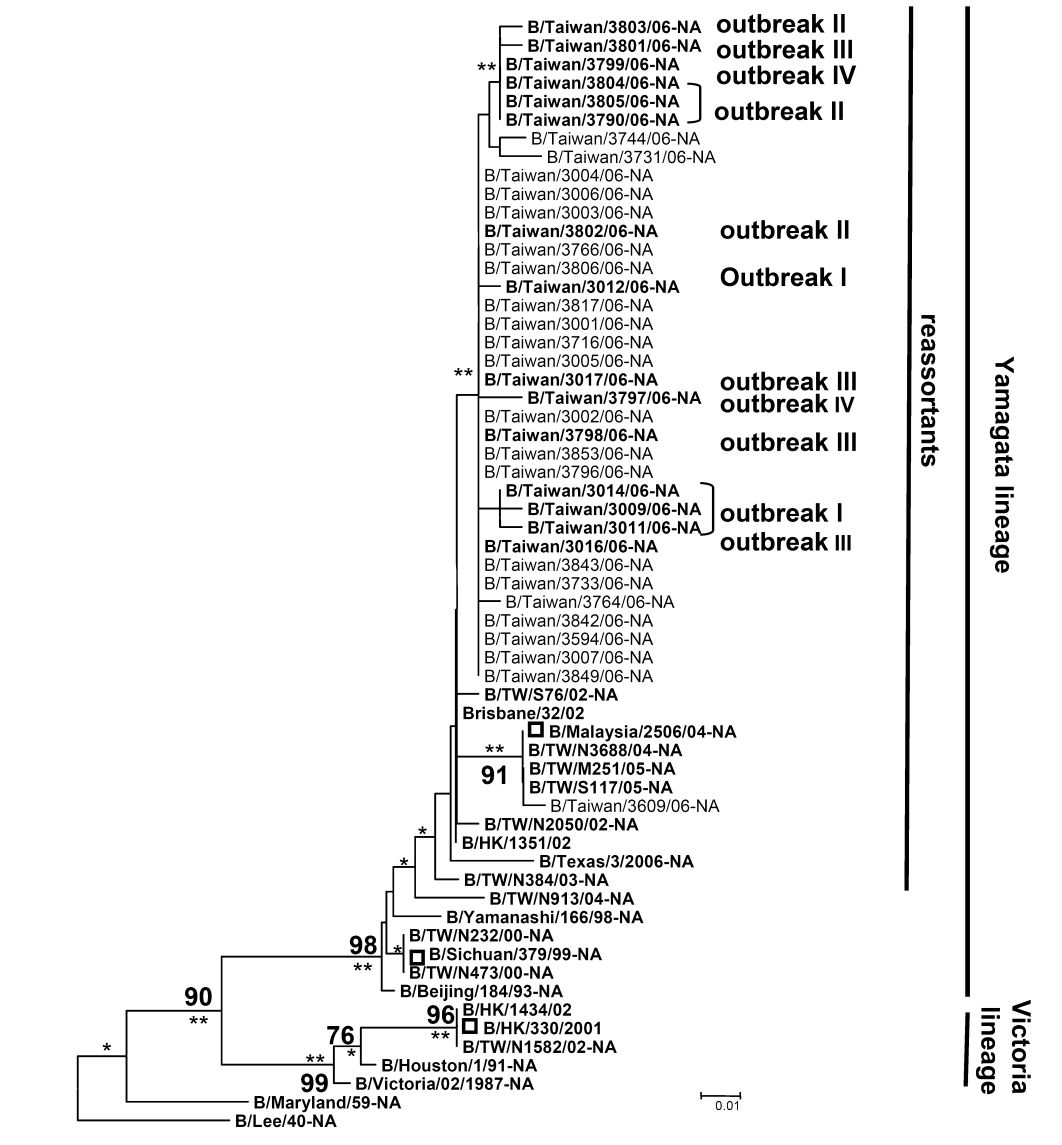
**Phylogenetic analysis of NA genes from influenza B virus isolates obtained between October 2006 and February 2007**. Dendrogram of 37 influenza B strains and 23 GenBank reference strains is based on 251 nucleotides (33 to 283) of the NA gene (neighbor-joining method with DNASTAR distance measure program; MEGA, Version 3.0). The linear unrooted phylogenetic dendrograms present the B/Lee/40 sequence as an outgroup. Branch length indicates genetic distance according to indicated distance scale (0.01, 1% difference). Bootstrap analysis percentages (n = 1000) for each branch are indicated. The statistical p-values were labeled on each node (*P < 0.05 and **P < 0.01).

According to results obtained from phylogenetic analyses of nearly full-length HA nucleotide sequences for the HA1 subunit (1100 nt) of influenza B virus isolates from 39 patients including 38 children and 1 adult (B/Taiwan/3842/06), all isolates clustered with B/Malaysia/2506/04-HA with a bootstrap value of 96 (P < 0.01) (Figure 3). B/Malaysia/2506/04 has been identified by the WHO as the primary vaccine strain for the 2006–2007 flu season in the northern hemisphere. Note that 4 variants (cluster I) in outbreak II clustered on a single branch with a bootstrap value of 79 and statistical p value < 0.01 (Figure 3). In terms of NA genes, all 37 Taiwanese isolates were identified as belonging to the B reassortant lineage (P < 0.05) except two isolates were not PCR amplified (Figure 4).

### Variation in HA and NA Amino Acid Sequences

Compared to amino acid residues 22–234 of the B/Malaysia/2506/04 HA protein, most of the Taiwanese isolates had only 2–3 changes. Exceptions included 1 strain from outbreak I (B/Taiwan/3009/06, 9 amino acid changes) and 4 variants (cluster I) from outbreak II (B/Taiwan/3802/06, B/Taiwan/3804/06, B/Taiwan/3805/06, and B/Taiwan/3790/06, with a combined total of 14 amino acid changes) (Table 1). Conserved region results indicate that amino acid residues 16–77 of the B/Malaysia/2506/04 NA protein originated in the previous season's Yamagata lineage strain (data not shown).

**Table 1:** Variations in HA amino acid sequence in recent influenza B viruses of Victoria lineage in Taiwan during 2006.10-2007.02 influenza season.

		Amino acid position																																
Outbreak	Virus strain(s)	2 4	2 5	4 8	4 9	7 9	8 0	8 3	1 0 0	1 1 6	1 2 1	1 2 7	1 2 8	1 2 9	1 3 4	1 3 5	1 4 6	1 5 4	1 6 4	1 7 6	1 8 2	1 8 6	1 8 9	1 9 7	1 9 9	2 0 4	2 1 4	2 1 6	2 1 7	2 1 8	2 2 0	2 2 1	2 2 8	2 3 0

Reference strains	B/Malaysia/2506/04	V	N	E	T	A	R	R	D	H	T	A	E	N	S	Y	V	A	D	E	T	D	T	N	A	L	T	S	A	N	V	T	I	G
	B/Beijing/243/97			K			K				I			K	P			V						T	T									
	B/HK/330/2001			K			K			R	N			K	P				E					S	T									
	B/TW/N1582/02			K			K			R	N			E	P				E						T									
	B/TW/N384/03			K			K			R	N			E	P		I		E						T									
	B/TW/N3688/04														P										T									
	B/TW/M251/05											V			P										T									
	B/TW/S117/05	L													P										T									

I	B/Taiwan/3009/06				A	P			H				K		P	H				K				T	T									
	B/Taiwan/3012/06														P										T									
	B/Taiwan/3014/06														P										T									

II	B/Taiwan/3802/06														P						P	Y	A		T	F			V					V
	B/Taiwan/3804/06														P										T							G	T	V
	B/Taiwan/3805/06														P						P				T	F	A				K	A		V
	B/Taiwan/3790/06														P						P				T	F		Y						V
	B/Taiwan/3803/06														P										T									
	B/Taiwan/3791/06														P										T									
	B/Taiwan/3792/06														P										T									

III	B/Taiwan/3017/06		Y												P										T									
	B/Taiwan/3798/06														P										T									
	B/Taiwan/3801/06														P										T									

IV	B/Taiwan/3799/06														P										T					S				
	B/Taiwan/3800/06														P										T					S				
	B/Taiwan/3019/06														P										T					S				

sporadic	B/Taiwan/3594/06*														P										T									
	B/Taiwan/3609/06										A				P										T									
	B/Taiwan/3733/06														P									S	T									
	B/Taiwan/3806/06														P										T					S				
	B/Taiwan/3853/06														P										T					D				
	B/Taiwan/3001/06								I																									
	B/Taiwan/3007/06														P										T						M			

*: Sporadic variant strain the same as 11 isolates including B/Taiwan/3716/06, B/Taiwan/3744/06, B/Taiwan/3731/06, B/Taiwan/3764/06, B/Taiwan/3766/06, B/Taiwan/3796/06, B/Taiwan/3817/06, B/Taiwan/3842/06, B/Taiwan/3843/06, B/Taiwan/3849/06, and B/Taiwan/1567/07.

### HI Titers

We used a panel of 4 reference sheep antisera to test the HI titers of 10 influenza B isolates; results are presented in Table 2. The HI titers of the B/Malaysia/2506/04-sheep antiserum to the Taiwanese influenza virus B strains belonged to outbreaks III, IV and the sporadic influenza B cases ranged from 1,280 to 2,560. In contrast, only 640 to 1,280 HI titers of the B/Malaysia/2506/04-sheep antiserum were identified as belonging to outbreak II. The number of HI titers for the B/Hong Kong/330/01-sheep antiserum ranged from 160 to 640. The smallest numbers of HI titers were observed in the antisera against the two virus isolates belonging to the Yamagata lineage.

**Table 2:** Antigenic virus identification of different Influenza B viruses.

		HI Antigen Typing (titer)			
		
Outbreak	Patient ID Number	B/Malaysia/2506/04^a^	B/Hong Kong/330/01^b^	B/Shanghai/361/02^c^	B/Sichuan/379/99^c^
II	B/Taiwan/3802/06	640	320	10	10
	B/Taiwan/3803/06	640	160	10	10
	B/Taiwan/3805/06	1280	320	10	10

III	B/Taiwan/3798/06	1280	320	10	10

IV	B/Taiwan/3799/06	2560	320	10	10

Sporadic	B/Taiwan/3853/06	1280	320	10	10
	B/Taiwan/3716/06	1280	160	10	10
	B/Taiwan/3733/06	2560	640	10	10
	B/Taiwan/3594/06	2560	640	10	10
	B/Taiwan/3001/06	2560	320	10	10
	B/Malaysia/2506/04^a^	320			
	B/Hong Kong/330/01^b^		160		
	B/Shanghai/361/02^c^			1280	
	B/Sichuan/379/99^c^				160

^a^B reassortant lineage

^b^Victoria lineage

^c^Yamagata lineage

### Post-Vaccination Serum Antibody Responses

Pre- and post-immunized serum samples were collected from 5 volunteers who had received the influenza vaccine for purposes of evaluating HI titers; all 5 generated antibody titers between 160 and 640 to the virus variants in outbreak II (Table 3). Of those 5, 3 showed 4 to 8-fold increases in HI antibody titers. The seroconversion factor was 1 for the patient diagnosed as immunologically incompetent. Another patient who reported to have received very few influenza vaccinations in the preceding ten years had exceptionally low antibody titers in his pre-immunized serum but an acceptable number following immunization.

**Table 3:** Comparison of Antibody Results and Seroconversion Factor for Reassortant Influenza B Viruses

		HI antibody results (titer)															
							
Outbreak	Patient ID Number	P004		P009		P109		P139		P182		PC^b^	Seroconversion factor^c^				
								
		pre^d^	post^e^	pre	post	pre	post	pre	post	pre	post		P004	P009	P109	P139	P182
II	B/Taiwan/3802/06	80	320	80	640	40	160	160	160	10	640	2560	4	8	4	1	64
(Cluster I)	B/Taiwan/3805/06	80	320	80	640	80	160	160	160	10	640	2560	4	8	2	1	64

III	B/Taiwan/3798/06	20	160	40	160	20	80	40	40	10	160	1280	8	4	4	1	16

	B/Taiwan/3594/06	40	320	40	320	40	320	80	80	10	160	5120	8	8	8	1	16
sporadic	B/Taiwan/3853/06	20	160	80	160	40	160	80	80	10	160	1280	8	2	4	1	16
	B/Taiwan/3001/06	20	160	40	320	20	80	40	40	< 10	80	2560	8	8	4	1	8

	PC^a ^(B/Malaysia)	40	160	80	160	20	80	40	40	10	80	640	4	2	4	1	8

^a^Positive control (B/Malaysia/2506/04) antigen (CDC, Atlanta, USA).

^b^Positive control (B/Malaysia/2506/04) reference sheep antiserum (CDC, Atlanta, USA).

^c^Seroconversion factor represents the number of fold-increase in Serum HI titer post-vaccination.

^d^Pre-immune.

^e^Post-immune.

## Discussion

At least three distinct variants of influenza B virus with origins in China have been collected from Taiwanese patients since 2002: B/Shanghai/361/2002-like (Yamagata lineage), B/Hong Kong/330/01-like (Victoria lineage), and B/Hong Kong/1351/02-like (B reassortant with Victoria-like HA and Yamagata-like NA genes) [19,21,22]. We characterized influenza viruses isolated form both children and adults living in north Taiwan between October 2006 and February 2007 for purposes of analyzing the antigenic properties and phylogenetic relationships of HA and NA genes from the influenza B virus isolates. Our results suggest the increasing reappearance of the VIC-HA/YAM-NA reassortant influenza B viruses during this period, with most outbreaks affecting children aged 6 to 8 years (Figures 1 and 2).

A total of 39 clinical isolates of reassortant influenza B viruses were used for molecular evolutionary analyses of HA and NA genes. Our results show that the HA genes belonged to the B/Victoria/2/87 lineage (Figure 3). We also found evidence of four outbreaks of reassortant influenza B viruses in the B/Victoria/2/87 lineage among elementary school-aged children living in Taiwan's Yilan County. Note the 4 variants (cluster I) in outbreak II that are clustered on a single branch in Figure 2–supported by a bootstrap value of 79%. The statistical P-value (P < 0.01) indicate the cluster strains for significance. These viruses were genetically similar to the B/Malaysia/2506/04 vaccine strain that was recommended as the vaccine candidate for the 2006–2007 flu season in the northern hemisphere. Results from sequence analyses of NA genes from the 37 Taiwan isolates indicate that they all belonged to the B/Yamagata/16/88 lineage (Figure 4)–that is, they clustered with two strains belonging to the B reassortant lineage, the B/Malaysia/2506/04 vaccine strain and B/Hong Kong/1351/02.

Compared to amino acid residues 22–234 of the B/Malaysia/2506/04 HA protein, most of the Taiwanese isolates had only 2–3 changes. Exceptions included 1 strain from outbreak I with 9 changes and 4 variants (cluster I) from outbreak II with 14 changes (Table 1). Genetic variation in this highly variant region suggests a potential for these reassortant variants to cause future outbreaks.

We performed HI tests for the identification of HA in circulating influenza B viruses. Our results show that the majority of circulating influenza B viruses (i.e., B/Malaysia/2506/04-like viruses) collected between October 2006 to and February 2007 belonged to the B/Victoria/2/87 lineage (Table 2). The data also indicate that the lowest HA titers were observed with sheep antisera against the other two isolates belonging to the Yamagata lineage. We used a panel of serum samples from 5 patients who received influenza vaccinations to test their ability to generate protective immunity against influenza B variants isolated from young children living in Yilan county, using seroconversion factor as our criteria. Of the 5 patients, 4 showed a minimum of 4-fold increases in HI titers post-vaccination; the seroconversion factor was 1 for the fifth patient, who was later diagnosed as immunologically incompetent (Table 3). These results suggest that the current influenza vaccine is still effective in stimulating protective immunity against the influenza B variants that we isolated [26-28]. Previously, Jian et al. reported a group of influenza B viruses isolated in Taiwan during the 2004–2005 and 2006–2007 epidemics [29]. In this study, we identified a group of children, lived in the northern regions of Taiwan, infected with influenza B virus variant. Most of them had severe headache symptoms when they had the virus infection. This has not been found by Jian et al. In addition, we have performed "hemagglutinin inhibition (HI) test" in our study (Table 2 and 3) which have never been reported by Jian et al.

The gene variable analysis of influenza viruses can provide information for epidemic and pandemic outbreak surveillance and determination of vaccine strain selection. In this study, we have shown data suggesting that the contemporary vaccine was still effective in stimulating protective immunity against the influenza B variants that we isolated. Therefore, continuous monitoring of emerging influenza B virus variants is vital to successful control not only locally, but also internationally.

## Competing interests

The authors declare that they have no competing interests.

## Authors' contributions

LYM performed the analysis of the data and drafted the manuscript. WSF and LCM revised the manuscript and did supplementary analysis. CKH, CYJ and LWT participated in the design of the study. CYMA participated in the design, revised the manuscript and coordination of the study. They all approved the final version of the manuscript.
